# Trematode diversity reflecting the community structure of Danish freshwater systems: molecular clues

**DOI:** 10.1186/s13071-020-04536-x

**Published:** 2021-01-12

**Authors:** Yajiao Duan, Azmi Al-Jubury, Per Walter Kania, Kurt Buchmann

**Affiliations:** Laboratory of Aquatic Pathobiology, Department of Veterinary and Animal Sciences, Faculty of Health and Medical Sciences, University of Copenhagen, Via Stigbøjlen 7, 1870 Frederiksberg C, Copenhagen Denmark

**Keywords:** Trematode, Snails, Diversity, Molecular identification, Freshwater lakes

## Abstract

**Background:**

Digenean trematodes are parasitic platyhelminths that use several hosts in their life cycles and are thereby embedded in various ecosystems affected by local environmental conditions. Their presence in a habitat will reflect the presence of different host species and, as such, they can serve as ecological indicators. Only limited information on the occurrence of trematodes and their link to other trophic levels in the Danish freshwater ecosystems is currently available.Therefore, the main aim of the present study was to increase our knowledge in this field.

**Methods:**

Snails were sampled from 21 freshwater lakes in Denmark, following which shedding procedures were performed, cercariae were recoved and the released parasites were identified using molecular tools (PCR and sequencing).

**Results:**

A total of 5657 snail hosts belonging to ten species were identified, revealing a highly diverse parasite fauna comprising 22 trematode species. The overall trematode prevalence was 12.6%, but large variations occurred between host species. The snail host *Lymnaea stagnalis* showed the highest prevalence and also exhibited the highest diversity, accounting for 47.6% of the species richness.

**Conclusions:**

This survey contributes updated information on parasite–host relations and compatibility and may assist in describing the ecological structure of the investigated Danish freshwater ecosystems. 
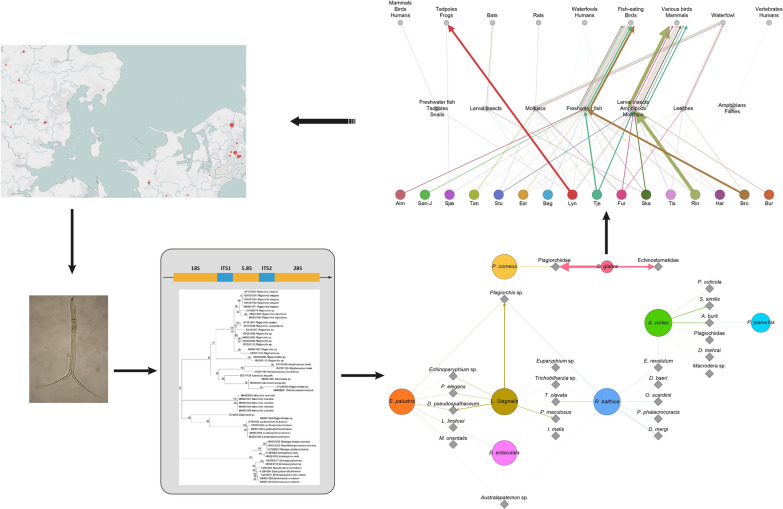

## Background

Trematodes, dominated by the digeneans, comprise a group of abundant and ubiquitous parasitic metazoans with high diversity and more than 18,000 nominal species [1]. These metazoans utilize multi-host systems to complete their complex life cycles, often with a mollusc as the first intermediate host [2–4]. Details on the associative life-cycle patterns between 1000 digenean species and their mollusc hosts have been recorded [5]. Other invertebrates and vertebrates may play a role as the second intermediate host, whereas the definitive hosts are often vertebrates, such as various fishes, amphibians, reptiles, birds and mammals [6]. The trematode life cycles involve intricate transmission patterns adapted to the habitat, the abiotic and biotic conditions and especially the different host species present [7].

Due to the obligate relation to free-living organisms in the ecosystem, these parasites may serve as bioindicators and used to indicate presence of obligate hosts, environmental conditions and disease risks under various conditions (climate changes, anthropogenic impacts) [2, 8–12]. This potential frames the importance of conducting continuous surveys of trematode occurrence in the freshwater environment in any region. However, due to the characteristics of adult digeneans, often hidden in their hosts and with uneven spatial distributions, it is relatively difficult to detect and monitor that stage. In contrast, sampling of the mollusc host in a habitat creates a method for efficient biomonitoring of their larvae. Incubation of an infected snail in the laboratory allows shedding of cercariae and thereby detection of a parasite at several trophic levels and hosts in the environment. The adults in the final hosts possess characteristic structures, but the larval stages related to the intermediate mollusc host (e.g. cercariae, rediae, sporocysts) and even metacercariae in the second intermediate host are generally more difficult to identify [13]. With the advent of molecular tools, such as PCR and subsequent sequencing of selected genes, it is now possible to elucidate the occurrence and diversity of various trematodes species in freshwater systems simply by sampling the mollusc host, recovering the released cercariae, isolating DNA, performing PCR and obtaining sequence information. Selected parts of the parasite genome, such as the nuclear ribosomal DNA regions comprising* 18S*,* ITS1*,* 5.8S*,* ITS2*,* 28S* and the mitochondrial cytochrome* c* oxidase subunit genes (*cox*1 and *cox*2) genes may be used to identify trematode species across a broad taxonomic range [14]. Diagnosis can be done by performing BLAST analysis against well-described species in GenBank.

Almost one century ago, the limnologist Wesenberg-Lund investigated the occurrence of cercariae in snails in a few Danish freshwater systems [15]. However, with a few exceptions, the link between the cercariae and other organisms in the environment was at that time poorly known. In addition, the identity of species of the recovered cercariae and their connection to other hosts in the ecosystem was in most cases not known. By using a molecular approach it is now possible to supplement our knowledge on the fauna of trematodes in Danish freshwater lakes [4, 16]. The present study describes the diversity of trematodes in 21 Danish freshwater lakes (geographically representing a major part of the national territory) by investigating ten species of molluscs and their released cercariae. By using current knowledge on transmission pathways, we obtained information on second intermediate and definitive host occurrence and trophic interactions in the ecosystems.

## Methods

### Field sampling

Samples of snails were recovered from a total of 21 selected freshwater lakes (sø) in Zealand (eastern part of Denmark) and Jutland (Western part of Denmark). The precise locations of the lakes were determined using the global positioning system (Table 1), and samplings were conducted from March 2019 to September 2019. Snails were collected by hand in shallow water areas, from stones and aquatic plants, placed in 1-l containers in cooled transport boxes and transferred (maximum 4 h transport time) to the university laboratory (Frederiksberg, Copenhagen area) for subsequent shedding procedures. In order to evaluate the effect of season (sampling date) on infection, three lakes (Bagsværd sø, Furesø and Esrum sø) were sampled several times between March and September 2019.

**Table 1:** Location, coordinates and surface area of lakes investigated during the survey conducted in Zealand and Jutland

Lake	Abbreviation	Location	Coordinates	Surface area (km^2^)
Ringen Sø	Rin	Zealand	55°37'56.1894", 12°4'55.1454"	0.007
*Bagsværd Sø*	Bag	Zealand	55°46'16.0566", 12°27'39.6864"	1.21
Utterslev mose	Utt	Zealand	55°43'5.3826", 12°30'28.0902"	2.00
Søndersø (Zealand)	Søn-Z	Zealand	55°46'34.8738", 12°20'58.6242"	1.44
Sjælsø	Sjæ	Zealand	55°52'4.1628", 12°26'27.3222"	2.93
Haraldsted Sø	Har	Zealand	55°29'8.3214", 11°47′ 48.0258"	2.00
*Furesø*	Fur	Zealand	55°47'53.682′′ 12°25′5.9838′′	9.40
Farum Sø	Far	Zealand	55°48'11.9268", 12°21'51.7998"	1.20
*Esrum Sø*	Esr	Zealand	56°0'5.1624", 12°22'23.1054"	17.29
Buresø	Bur	Zealand	55°49'30.9498", 12°13'6.5424"	0.76
Tissø	Tis	Zealand	55°34'38.6178", 11°17'16.2672"	12.30
Lyngby Sø	Lyn	Zealand	55°46'27.4866", 12°29'10.2726"	0.57
BrommeLille Sø	Bro	Zealand	55°28'52.2402", 11°30'51.5736"	0.125
Skanderborg Sø	Ska	Jutland	56°1'12.45", 9° 55'34.089"	6.32
Stubbe Sø	Stu	Jutland	56°15'28.2882", 10°41'15.9972"	3.76
Mossø	Mos	Jutland	56°2'23.139", 9°46'24.3696"	16.50
Tange Sø	Tan	Jutland	56°19'43.9062", 9°34'56.445"	5.40
Tjele langsø	Tje	Jutland	56°32'11.5902", 9°38'39.372"	3.90
Halle Sø	Hal	Jutland	55°59'14.388", 9°28'27.4656"	0.31
Søndersø (Jutland)	Søn-J	Jutland	56°26'22.9878", 9°24'47.5596"	1.41
Almind Sø	Alm	Jutland	56°8'59.553", 9°32'37.4382"	0.53

Lakes written in italics were involved in the seasonal variation study and sampled several times from March to September. Other lakes were only sampled once

### Cercarial shedding from snails

Once in the laboratory, the sampled snails were rinsed in de-chlorinated municipal water for removal of surface-adhering organisms (preventing interference of false positives), whereafter they were identified to species by shell morphology [17]. Individual snails were placed at room temperature in 100-ml beakers containing 50 ml of 0.45-µm filtered lake water (Filtropur; Sarstedt, Nümbrecht, Germany) [18] and incubated for 8 h in daylight. The beakers were subsequently examined under a stereomicroscope (magnification ×7–40; Leica Microsystems GmbH, Wetzlar, Germany) to record presence/absence of cercariae. Infected snails were characterized as “positive” and non-infected snails as “negative”, following which the cercarial type was determined [19–22]. Recovered cercariae were preserved in 96% ethanol (Kemetyl A/S, Køge, Denmark) for subsequent molecular analysis [23].

### Molecular identification of cercariae

Individual ethanol-fixed cercariae were separated and placed in Eppendorf tubes (Axygen®, Sorenson BioScience, Salt Lake City, UT, USA), following which the ethanol was evaporated (Eppendorf ThermoMixer® Comfort; Eppendorf, Hamburg, Germany). DNA from each specimen was then isolated following lysis (using a guanidine thiocyanate lysis buffer) (Tkach and Pawlowski [24]). DNA was purified from the PCR mixture using Illustra™ GFX™PCR and Gel band purification kit (VWR International, Søborg, Denmark). DNA concentration and purity were measured using a Nanodrop 2000 spectrophotometer (Saveen & Werner ApS, Jyllinge, Denmark). The complete* ITS1*–*5.8S*–*ITS2* regions of the rDNA were amplified using forward primer BD1 (5′-GTC GTA ACA AGG TTT CCG TA-3′) and reverse primer BD2 (5′-TAT GCT TAA ATT CAG CGG GT-3′) [25] under PCR cycling conditions as previously described [26]. Each sequence obtained from the PCR conducted was subjected to BLAST analysis at NCBI (National Center for Biotechnology Information). All sequences were submitted to GenBank and obtained accession numbers.

### Evolutionary analysis by maximum likelihood method

Evolutionary analyses were conducted in MEGA X [27]. Clustal W was used to align the sequences, and the model General Time Reversible G+I using the Akaike criterion was the best fitting model of the 24 models tested by MEGA X. Therefore, the evolutionary history was inferred by using the maximum likelihood method and general time-reversible model. The tree with the highest log likelihood (− 31524, 07) is shown in the Electronic Supplementary Material. The percentage of trees in which the associated taxa clustered together is shown next to the branches. Initial tree(s) for the heuristic search were obtained automatically by applying the neighbor-joining and BioNJ algorithms to a matrix of pairwise distances estimated using the maximum composite likelihood (MCL) approach, and then selecting the topology with the superior log likelihood value. A discrete gamma distribution was used to model evolutionary rate differences among sites (5 categories (+ G, parameter = 15143)). The rate variation model allowed for some sites to be evolutionarily invariable ([+ I], 9.91% sites). This analysis involved 89 nucleotide sequences. There were a total of 1960 positions in the final dataset.

### Data analysis

Raw data were entered into a Microsoft Excel (Microsoft Corp., Redmond, WA, USA) spreadsheet, and descriptive statistics were used to summarize the data. A Chi-square test was used to assess any significant differences with regard to prevalence in different seasons. Pearson's correlation analysis was used to assess correlation between the number of trematode species and snail species in each lake. The data were analyzed statistically using the RStudio software program (v.3.44; R Foundation for Statistical Computing, Vienna, Austria). The software Tableau (v.2019.3) was applied for community structure (biodiversity) analysis, and Gephi v.0.9.2 was used to illustrate the interconnection between trematode, infected snail and parasite–host transmission pattern. Statistical significance was set at *P* < 0.05 for all statistical tests.

## Results

### Field sampling

**Fig. 1:**
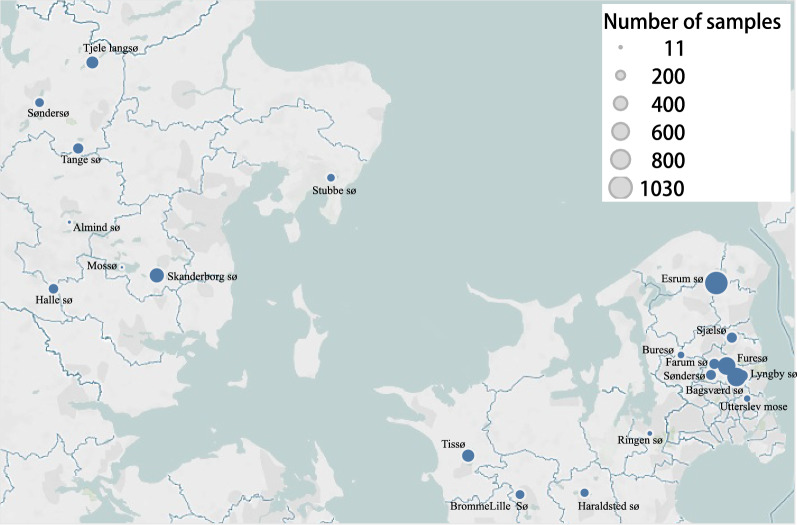
Sampling locations in Danish freshwater lakes with indications of sample size (diameter of circles)

Sample sizes at the 21 different locations are indicated in Fig. 1. Large samples were recovered in Esrum sø (1030), Furesø (852), Bagsværd (695) and Skanderborg sø (405), whereas relatively small samples were collected in Utterslev mose (81), Almind sø (18) and Mossø (11). Sixteen lakes were positive with regard to trematode infection (cercarial shedding), whereas the remaining five locations were negative for parasites.

### Prevalence of infection at different locations

We examined a total of 5657 individual freshwater snails belonging to three families and ten different species: Lymnaeidae (*Lymnaea stagnalis*,* Radix balthica*,* Radix auricularia*,* Stagnicola palustris* and *Omphiscola glabra)*; Planorbidae (*Planorbarius corneus*,* Planorbis planorbis* and *Anisus vortex*); and Bithyniidae (*Bithynia leachii* and *Bithynia tentaculata*). The samples were dominated by *Anisus vortex* (1402),* Radix balthica* (1200) and *Lymnaea stagnalis* (1173). Of the 5657 snails examined, 715 (eight species) released cercariae, resulting in an overall prevalence of 12.6%. The most prevalent snail overall was *Lymnaea stagnalis* (18.2%), followed by *Radix balthica* (14.4%). Seven snail species were collected in Furesø and six species in Bagsværd sø, Esrum sø and Skanderborg sø (Table 2), with only one snail species collected from each of the other lakes, such as the artificial lake Ringen sø. Sixteen lakes were positive and five lakes were negative for trematode infection, but some lakes showed a relatively high prevalence of infection. Thus, Ringen sø showed a 46.7% prevalence of trematode infection, Bromme Lille sø 29.0% and Tjele Langsø 27.9%. A number of other lakes showed an intermediate prevalence level of between 5 and 23% (Almind sø, Bagsværd sø, Furesø, Lyngby sø, Tissø, Skanderborg sø), and a few lakes showed a low prevalence of < 5% (Sjælsø, Haraldsted sø, Buresø). Since the 21 lakes were monitored at different sampling dates, it was considered important to test for a possible occurrence of a seasonal variation which could affect the overall data set (Table 3). To this end, we sampled three different lakes as an internal reference (Bagsværd sø, Furesø and Esrum sø) three times from March to September. During these samplings, we collected a total of 119 snails in March, 900 in July and 386 in September. A Chi-square test showed that prevalence of infection on these sampling dates was significantly different (*P* < 0.05), with the highest prevalence appearing in September (48.96%) and lowest prevalence showed in March (1.68%).

**Table 2:** Total number of snails (ten snail species) sampled in 21 lakes

Snail	Infection level	Lakes^a^																					Total
		Rin	Bag	Utt	Søn-Z	Sjæ	Har	Fur	Far	Esr	Bur	Tis	Lyn	Bro	Stu	Ska	Mos	Tan	Tje	Hal	Søn-J	Alm	
Lymnaeidae																							
* Lymnaea stagnalis*	No. snails examined	90	54	21	16	5	3	163	9	207	2	–	–	25	97	82	6	104	214	41	27	7	1173
	No. snails infected	42	5	0	0	0	0	49	0	13	0	–	–	0	7	32	0	4	61	0	0	0	213
	Prevalence (%)	46.7	9.3	0	0	0	0	30.1	0	6.3	0	–	–	0	7.2	39.0	0	3.8	28.5	0	0	0	18.2
* Radix balthica*	No. snails examined	–	303	44	75	22	101	18	15	111	–	–	–	110	13	21	3	49	73	140	93	9	1200
	No. snails infected	–	30	0	0	0	1	14	0	42	–	–	–	45	0	4	0	7	19	0	9	2	173
	Prevalence	–	9.9	0	0	0	0.99	77.8	0	37.8	–	–	–	41.0	0	19.0	0	14.3	26.0	0	9.7	22.2	14.4
* Radix auricularia*	*No. examined*	*–*	*–*	*–*	*–*	9	–	–	25	–	*–*	*–*	*–*	*–*	*–*	*–*	*–*	*–*	*–*	*–*	*–*	*–*	34
	*No. infected*	*–*	*–*	*–*	*–*	0	–	–	0	–	*–*	*–*	*–*	*–*	*–*	*–*	*–*	*–*	*–*	*–*	*–*	*–*	0
	*Prevalence (%)*	*–*	*–*	*–*	*–*	0	–	–	0	–	*–*	*–*	*–*	*–*	*–*	*–*	*–*	*–*	*–*	*–*	*–*	*–*	0
* Stagnicola palustris*	*No. snails examined*	*–*	72	–	3	–	2	28	–	389	42	183	33	–	–	166	–	–	–	–	–	–	918
	*No. snails infected*	*–*	18	–	0	–	1	4	–	62	2	10	4	–	–	11	–	–	–	–	–	–	112
	*Prevalence (%)*	*–*	25.0	–	0	–	0.5	14.3	–	15.9	4.8	5.5	12.1	–	–	6.6	–	–	–	–	–	–	12.2
* Omphiscola glabra*	*No. snails examined*	*–*	*–*	*–*	*–*	*–*	*–*	6	–	–	–	–	–	–	–	8	–	–	–	–	–	2	16
	*No. snails infected*	*–*	*–*	*–*	*–*	*–*	*–*	2	–	–	–	–	–	–	–	4	–	–	–	–	–	2	8
	*Prevalence (%)*	*–*	*–*	*–*	*–*	*–*	*–*	33.3	–	–	–	–	–	–	–	50	–	–	–	–	–	100	50
Planorbidae																							
* Planorbarius corneus*	No. snails examined	–	199	16	44	–	1	208	–	40	27	*–*	58	–	–	17	1	45	–	–	31	–	687
	No. snails infected	–	1	0	0	–	0	57	–	0	0	*–*	3	–	–	3	0	4	–	–	0	–	68
	Prevalence (%)	–	0.5	0	0	–	0	27.4	–	0	0	*–*	5.2	–	–	17.6	0	8.9	–	–	0	–	9.9
* Planorbis planorbis*	No. snails examined	–	–	–	–	–	–	–	–	–	14	113	–	–	–	–	–	–	–	–	–	–	127
	No. snails infected	–	–	–	–	–	–	–	–	–	0	5	–	–	–	–	–	–	–	–	–	–	5
	Prevalence (%)	–	–	–	–	–	–	–	–	–	0	4.4	–	–	–	–	–	–	–	–	–	–	3.9
* Anisus vortex*	No. snails examined	–	46	–	59	169	–	422	157	262	–	–	158	–	6	111	1	11	–	–	–	–	1402
	No. snails infected	–	0	–	0	4	–	59	0	10	–	–	51	–	0	5	0	1	–	–	–	–	130
	Prevalence (%)	–	0	–	0	2.4	–	14.0	0	3.8	–	–	32.3	–	0	4.5	0	9.0	–	–	–	–	9.3
Bithyniidae																							
* Bithynia leachii*	No. snails examined	–	–	–	–	–	27	7	–	–	–	–	–	–	–	–	–	–	–	–	–	–	34
	No. snails infected	–	–	–	–	–	0	0	–	–	–	–	–	–	–	–	–	–	–	–	–	–	0
	Prevalence (%)	–	–	–	–	–	0	0	–	–	–	–	–	–	–	–	–	–	–	–	–	–	0
* Bithynia tentaculata*	No.snails examined	–	21	–	–	–	–	–	–	21	3	–	1	20	–	–	–	–	–	–	–	–	66
	No. snails infected	–	4	–	–	–	–	–	–	1	0	–	1	0	–	–	–	–	–	–	–	–	6
	Prevalence (%)	–	19.0	–	–	–	–	–	–	4.8	0	–	100	0	–	–	–	–	–	–	–	–	9.1
Total prevalence (%)		53.5	8.3	0.0	0.0	2.0	1.5	21.7	0.0	12.4	2.3	5.1	23.6	29.0	6.0	14.6	0.0	7.7	27.9	0.0	6.0	22.2	12.6

Sampling from March to September 2019, with the number of examined snails, infected snails, overall prevalence of trematode infections (% of infected snails in the overall samples)

^a^See Table1 for details on lakes

**Table 3:** Seasonal variation of prevalence in three lakes (Bagsværd sø, Furesø and Esrum sø)

Infection level	Sampling dates							*χ*^2^ (*P*)
	March	April	May	June	July	August	September	
Number of snails examined	119	544	191	203	900	432	386	
Number of snails infected	2	76	5	33	93	117	189	
Prevalence of infection (%)	1.68	14.0	2.62	16.26	10.33	27.08	48.96	93.66 (< 0.001)

*χ*^2^: Chi-square test

### Trematode fauna

Sequences of the rDNA locus* ITS* of the different parasite isolates were submitted to GenBank. In order to identify the trematodes, both percentage identity obtained from a BLAST search of GenBank and a phylogenetic analysis were used. Sequenced specimens all belonged to class Digenea (GenBank acc. nos. MW000945 to MW001148), and of these 52 sequences were unique. The* ITS1*–*5.8S*–*ITS2* part of these 52 sequences were subjected to a phylogenetic analysis together with 34 other digenean species. Three species of the sister class Aspidogastrea were used as an outgroup (marked in bold on Additional file 1: Fig. S1). A total of 22 trematode species belonging to nine families were recorded (Additional file 2: Table S1). The diversity of trematode species differed markedly between snail hosts. Some species of snails exhibited a rich trematode fauna. *Lymnaea stagnalis* was found to be infected by ten species and showed the highest overall prevalence of infection (18.2%). In some snail species, such as *Planorbis planorbis* and *Bithynia tentaculata*, only one trematode species was recovered.

The host snail *Lymnaea stagnalis* was found to be infected by several types of digeneans belonging to the family Plagiorchiidae. Species within the genus *Plagiorchis* were the most frequently recovered, showing an overall prevalence of 5.0%. This species was also prevalent in spring samples despite a generally lower infection rate in this season. Three species with a zoonotic potential were recovered, including *Isthmiophora melis* (found in Skanderborg sø)*, Trichobilharzia* sp. (found in Bagsværd sø, Tjele langsø and Furesø) and *Metorchis orientalis* (found in Lyngby sø and Skanderborg sø)*.*

The parasites identified utilize a wide variety of second intermediate and definitive hosts (based on published data from the literature; Additional file 2: Table S1) falling within six transmission units. One of the entities are parasites utilizing fishes as the second intermediate host and fish-eating birds as the definitive host (seven species). This group of trematodes is mainly associated with *Radix balthica* and dominated the digenean fauna in BrommeLille sø. Another group uses molluscs as the second intermediate host and waterfowl as the definitive host (two species). A third group utilizes larval insects as second intermediate host and bats as the definitive host (one species), whereas a fourth group uses frogs (two species) or waterfowl (one genus) as the final host. The fifth group utilizes leeches as the second intermediate host and waterfowl as the definitive host (2 species). Finally, a sixth group comprises species with multiple intermediate and definitive hosts (four species), including larval insects, amphipods, molluscs, leeches and mammals.

### Parasites reflecting host occurrence in a lake

**Fig. 2:**
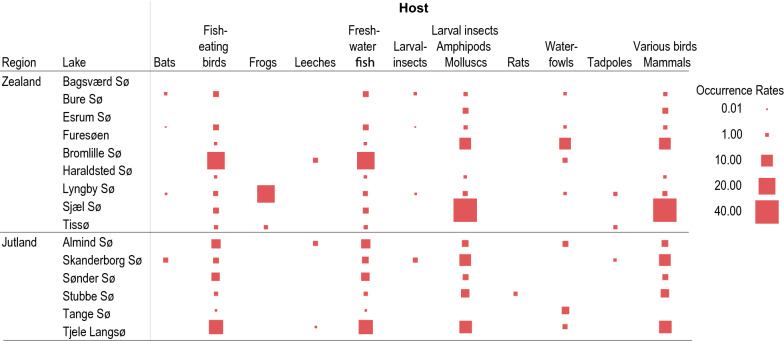
Indication of host type occurrence inferred by the trematode fauna in Danish freshwater lakes (Zealand and Jutland). Note: the size of the square (occurrence rates) indicates the proportion of the host type utilized by a corresponding trematode species in each lake. The proportion was calculated based on the raw data shown in Additional file 2: Table S1

Digeneans are relatively host specific, especially at the mollusc host level, and each species utilizes generally one or few related hosts at each stage of its life cycle. Based on published information on hosts and life cycles, we constructed a list of possible animal species present in each lake (Additional file 2: Table S1). Lakes in Zealand and Jutland did not differ in terms of host occurrence inferred from the trematode species present (Fig. 2). Freshwater fish, fish-eating birds, larval insects, amphipods, molluscs, various birds and mammals were host types deducted from the parasite lists in both the eastern and western locations. Primarily BrommeLille sø in Zealand and secondly Tjele langsø in Jutland were populated with parasites, indicating a high abundance of freshwater fish and fish-eating birds. Snails collected from Ringen sø shed parasites, indicating larval insects, amphipods and molluscs, birds and mammals. The presence of waterfowl, tadpoles, leeches and bats were indicated both in Zealand and Jutland. In Zealand (the eastern part of Denmark), a number of host types were indicated, with a higher host diversity in Lyngby sø (9 types), Esrum sø (7 types), Bagsværd sø (7 types) and Furesø (5 types). These were followed, in terms of host diversity, by Haraldsted sø, Tissø and Sjælsø. In Jutland (western part of Denmark) the highest number of host types were seen in Skanderborg sø (7 types) followed by Tjele langsø (6 types).

## Discussion

Investigations on the trematode fauna of freshwater snails in a certain habitat can contribute to our knowledge and understanding of the ecology of trematodes. A basic knowledge of life cycles with the inclusion of obligate intermediate and/or final host(s) will reflect the existence of other faunal elements in the habitat. The most recent study on Danish freshwater systems involving cercariae released by snails was conducted almost one century ago by Wesenberg-Lund [15] in selected Danish water bodies. Since then considerable research efforts have been invested in the elucidation of life cycles, and this knowledge in this field can be used to elevate our overall understanding of the freshwater biotope. In our study, we conducted a comprehensive and expanded investigation on cercariae released from various snail hosts in Danish lakes. This will serve as an updated record of the trematode fauna in Denmark, link the snail parasites to other trophic levels and facilitate evaluations of the freshwater systems involved. Recent studies in other European regions and in Lake Victoria in Afrtica [28] provide a valuable basis for comparison. Selbach et al. [29] examined 5347 snails sampled in German lakes belonging to six species which were infected with a total of 36 trematode species. Similar studies were conducted at five aquatic locations of Europe during the latest decades (Table 4). Our samplings comprised 5657 specimens of snails belonging to ten species within three families, and we found 715 snails infected with parasites, resulting in an overall prevalence of 12.6%. This level of prevalence and the number of species (22) in Danish lakes correspond well with several records from other freshwater bodies in Europe and Africa.

**Table 4:** Overview of comparable snail–trematode diversity and overall prevalence

Sampling sites	Number of snails	Number of snail species	Number of trematode species	Overall prevalence (%)	References
West coast of Eyjafjordur, North Iceland	1519	14	7	n/a	Sannia et al. [82]
Porma river basin, Spain	6291	1	3	15.50%	Manga-González et al. [83]
River and reservior, Poland	1510	n/a	6	32.60%	Jeżewski [84]
Two fishponds and one swamp in Czech Republic	2802	12	28	33.90%	Faltýnková [32]
Rivers, ponds and lakes in Germany	6403	15	29	4.90%	Faltýnková et al. [36]
Lake Konnevesi in central Finland	3628	n/a	24	26.30%	Faltýnková et al. [85]
29 lakes in Poland	10581	6	25	46.50%	Żbikowska [47]
Forest biocoenoses of Ukrainian Polissia Nature Reserve	n/a	14	26	n/a	Zhytova et al. [6]
Rivers Lippe and Rhine in North Rhine-Westphalia, Germany	682	1	20	12.90%	Schwelm et al. [86]
Interconnected Ruhr lake system, Germany	5347	6	36	19.60%	Selbach et al. [29]
21 freshwater lakes, Denmark	5657	10	21	12.60%	Present study

n/a, Not available

We selected a number of freshwater lakes for our investigation with the aim to cover both the western (represented by Jutland) and eastern (represented by Zealand) parts of Denmark. The intentions was to collect a high number of snails at all locations, but the sample sizes were low at some localities and on certain sampling dates (Almind sø and Mossø). Low sample sizes may be explained by the practical and logistic challenges associated with limited access to private property, physical inaccessibility, elevated water levels and inferior weather conditions, which in turn may bias observations when different lakes are compared. However, the numbers were sufficient to discuss the biological implications when finding a certain type (genus, species) of cercaria released from freshwater snails in a specific lake. Thus, the presence of obligate intermediate and final hosts is indicated if a parasite is found in the ecosystem. We do not at present have sufficient information on each lake’s history to draw conclusions on causative factors (abiotic, biotic, anthropogenic). However, it is known that both abiotic and biotic factors influence the occurrence of snails and parasites, that the pond area of eutrophic water bodies influences the occurrence of freshwater snails [30] and that *Bithynia tentaculata* is affected by human recreational transport (proximity to a boat launch) and eutrophication (anthropogenic land use, elevated nutrient concentrations) [31]. Future analyses should therefore include advanced and comprehensive physico-chemical lake data collection to allow advance the conclusions drawn from the present study. This may allow us to implement corresponding studies on how these factors theoretically could affect parasites in the infected snails in Danish lakes as well.

It is noteworthy that the minimal snail richness in Ringen sø was associated with a relatively high prevalence of trematode infection (46.7%). Corresponding high infection levels have been observed previously, such as a 47.1% prevalence of infection in *Lymnaea stagnalis* in the Czech Republic [32]. It may be that the local environmental conditions Ringen sø account for this high prevalence (46.7%). This leads to the speculation that this artificial and young lake (established 1977), which generally has a low biodiversity, provides optimal conditions for the propagation of a single snail species and a few associated parasite species following their first introduction with local mallards and swans.

We showed that the prevalence of infection showed a seasonal variation—at least based on the three representative Danish lakes sampled from March to September. Therefore, it is not reasonable to directly compare different lakes which were sampled at different time points. Our analyses will therefore not include overall correlations between abiotic conditions and infection levels, although Soldánová [33] did show that the infection probability of *L. stagnalis* was associated with levels of dissolved oxygen and pH of lakes.

The most abundant snail species in our study was *Anisus vortex* (sample size 1402), followed by *Radix balthica* (1200) and *Lymnaea stagnalis* (1173). The trematode diversity was highest in *Lymnaea stagnalis* (10 species), *Radix balthica* (10 species) and *Stagnicola palustris* (7 species); these results correspond partly to those of a previous study conducted by Selbach et al. [29] showing that snails within the genera *Radix* (Lymnaeidae) and *Gyraulus* (Planorbidae) accounted for almost 90% of the trematode diversity. Similar studies from Canada and Scandinavia also point to high prevalences and trematode diversity, especially in lymnaeids [34]. When evaluating the importance of snail hosts in Danish lakes, we show here that the three species *L. stagnalis*,* R. balthica* and *S. palustris* play a central role for trematode infections in the ecosystem.

A total of 22 trematode species belonging to nine families were found in ten species of snails. It may be argued that the trematode diversity may be influenced by the host sample size, a possibility referred in previous studies showing that a higher number of parasite species was collected from the more abundant snail species [29]. In the present study we collected almost equal numbers of the snails *R. balthica* and *L. stagnalis*, and both of these hosts carried ten digenean species, corresponding to data reported in a recent Norwegian study [35]. The central role of lymnaeids for trematode diversity in freshwater lakes was further framed by a high diversity (23 species) in *R. auricularia* in Germany [29], although a lower diversity may be found in the Czech Republic (three species) [32] and southeast Germany (four trematode species) [36].

In a German study *R. auricularia* harbored six trematode species of family Diplostomidae and contributed to a high diversity of trematode species [29], which is consistent with our study. In addition to identifying *Diplostomum baeri*, *D. mergi and Tylodelphys clavata*, the authors of that study also found *D. parviventosum and D. spathaceum*, compared with *Ornithodiplostomum scardinii*, *Posthodiplostomum cuticola* and *D. pseudospathaceum* released from *Radix balthica* in our study. Our study is the first to report the presence of *O. scardinii* cercariae, but the adult specimens have been isolated from infected birds in the Czech Republic and Poland [37].

Another investigation of biodiversity of trematodes in freshwater ecosystems of Europe [13] reported that species within the families Diplostomidae, Echinostomatidae and Schistosomatidae were the most frequently recorded. The most frequently recorded trematode species in these studies is *Diplostomum spathaceum*, which was recorded in the Czech Republic [38], UK [39], Finland [40], Lithuania [41], Poland [42], Russia [43] and Switzerland [44]. Another species, *Diplostomum pseudospathaceum*, was found in Switzerland [44], UK [45], Czech Republic [32], Finland [46], Poland [47], Lithuania [41] and Germany [36]. A third common species is *Echinoparyphium recurvatum* found in the Czech Republic [38, 48], Poland [47], Germany [36], Finland [49], Iceland [50], Ukraine [51] and Russia [43].

**Fig. 3:**
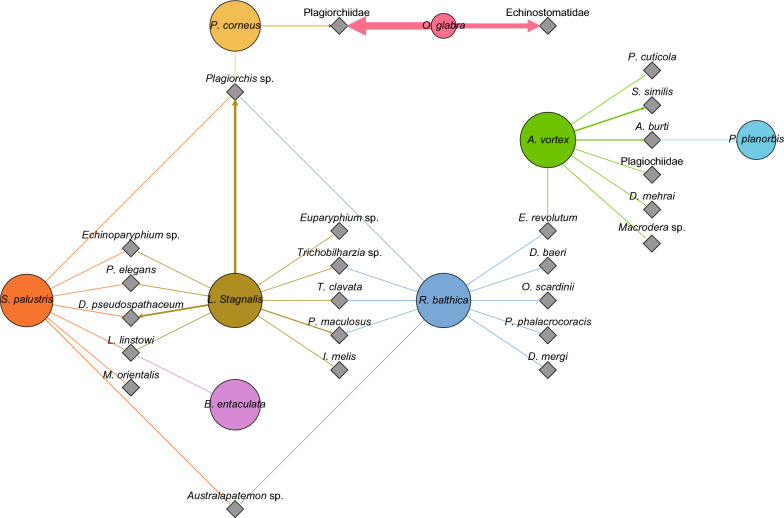
Graphical presentation of infected snail species and their interconnection through the trematode fauna in the investigated Danish freshwater lakes. Note:trematode species associated with particular hosts are indicated. Each circle represents one host snail species, with the area of each circle corresponding to the total number of snails sampled during the study. The thickness of the lines is proportional to the number of trematode species utilizing each snail species

The findings of our study in Denmark correspond to a certain extent to these trends. Our study shows that Diplostomidae (six species), Echinostomatidae (five species) and Plagiorchiidae (four species) were the most frequently recorded trematode families. Various species within the genus *Plagiorchis* were frequently isolated in the present study, corresponding to occurrence reported in Poland [47], Czech Republic [32], Finland [49] and Germany [36]. *Lecithodendrium linstowi* was the most frequently recorded species in our study (Fig. 3; Table 4).

**Fig. 4:**
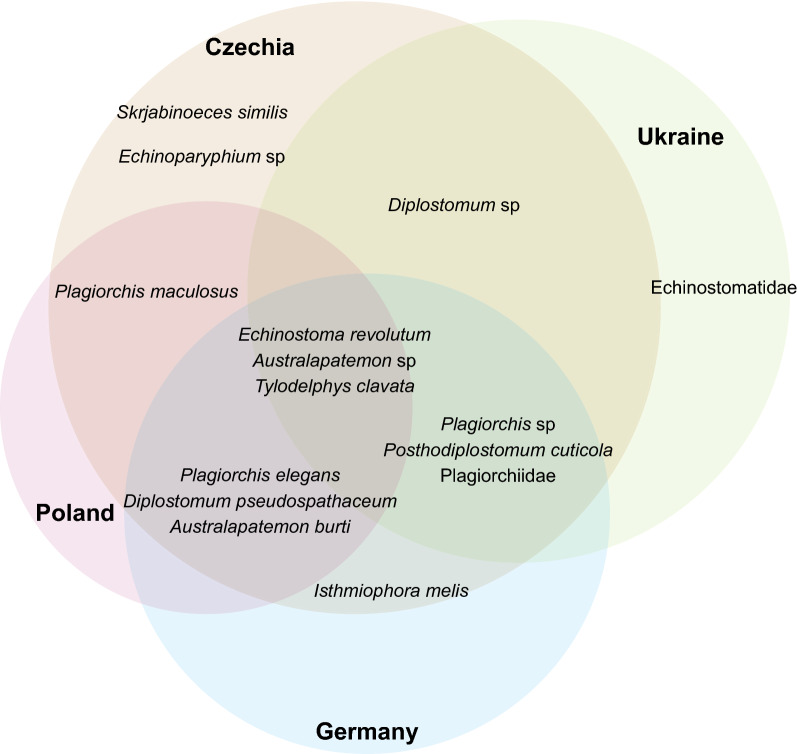
Comparison of trematode species recorded in four countries (Czech Republic [Czechia[), Germany, Ukraine and Poland) in Europe

Comparison of the latest Danish records with the results of studies on freshwater snail–trematode associations carried out in Europe since the beginning of the 20th century revealed some trends. Data from the Czech Republic indicate high a similarity in terms of trematode diversity, with 14 species (66.7%) of trematodes overlappingwith our study. This was followed by Germany (10 species, 47.6%), Ukraine (8 species, 38.1%) and Poland (7 species, 33.3%) [44] (Fig. 4). Three species, *Echinostoma revolutum*,* Australapatemon* sp. and *Tylodelphys clavata* were the most universally recorded parasites (Fig. 4).

In our study, three types of zoonotic trematodes were discovered, *Isthmiophora melis*, *Trichobilharzia* sp*.* and *Metorchis orientalis*, which parasitized *L. stagnalis, R. balthica* and *S. palustris,* respectively, all snail hosts belonging to the family Lymnaeidae (Table 5). The first intermediate host of *Isthmiophora melis* is *L. stagnalis* [52], and amphibians and fish serve as second intermediate hosts [53, 54]. In China, raw loach meat was reported to be a source of human infection, with adult specimens parasitizing the human intestine and causing diarrhea [55, 56]. *Isthmiophora melis* has not been reported in many countries, with the exception of Germany [57] and Czech Republic. Various bird schistosome species within the genus *Trichobilharzia* use only one intermediate host (pulmonate snails) [58–61]. Species within the genus *Radix* may serve as intermediate hosts for *Trichobilharzia* spp., but they are considered less specific hosts compared to *L. stagnalis* [62]*.* Cercariae shed from the snail may accidentially penetrate human skin, instead of their natural target (bird skin), and may within a few minutes after elicit cercarial dermatitis [63, 64]. Species within the genus *Trichobilharzia* have been reported in numerous locations including, among others Czech Republic [32], Poland [47], Finland [49], Russia [43] and Germany [36].

**Table 5:** Species of trematodes with a zoonotic potential and their hosts recorded in the present study.

Trematode		GenBank		Host species	References
Family	Species	Accession no.	Similarity (%)		
Diplostomidae	*Isthmiophora melis*	KT359584	100	*Lymnaea stagnalis*	Radev et al. [54]
Schistosomatidae	*Trichobilharzia* sp.	KP271014	99.8	*L. stagnalis *&* Radox balthica*	Haas et al. [63]
Opisthorchiidae	*Metorchis orientalis*	MK482053	97.9–98.3	*Stagnicola palustris*	Na et al. [70]

Digenean species within the family Opisthorchiidae have been less well studied in Europe, and data from our study adds to our knowledge on the presence and life cycle of *Metorchis orientalis.* Species of genus *Metorchis* have been previously recorded in Finland [65], and numerous human cases are known in Asia (e.g. China and Japan), where *Parafossarulus striatulus*,* Bulimus striatulus *[66–68] and *Bythinia* sp. [69] can act as the first intermediate host. Adult trematodes commonly inhabit the bile duct and gallbladder of mammals, including humans, causing hepatitis and, in severe cases, even death [70, 71]. Infection is acquired by eating raw freshwater fishes [72], and susceptible fish host species do occur in Denmark*.* It is therefore relevant to note that the local lymnaeid snails are potential hosts for this type of zoonotic parasites in Danish freshwater lakes.

Trematodes of the family Plagiorchiidae are ubiquitous parasites and widespread in different host species [73], as reflected by their common occurrence in Danish snails (Fig. 3). We recorded 12 lakes positive for genera within the family Plagiorchiidae. Based on the findings of the present study, it is clear that the genus *Plagiorchis* is prevalent in *Lymnaea stagnalis*, with a prevalence of up to 6.4% (for types within the genus *Plagorchis* which were not identified to the species level). For the two species *P. elegans* and *P. maculosus*, we found a prevalence of 1.5 and 2.8%, respectively (Additional file 2: Table S1). The four different species of snail hosts could indicate a relatively low host specificity (Fig. 3), but it must be emphasized that species identification was not achieved for these parasites in all cases. If an improved differential diagnostic technique for the separation of species could be applied, we may achieve a higher species resolution which could reveal a higher specificity. This group of parasites was also found in Ringen sø with only one snail species present. This is an artificial lake constructed 40 years ago and harbors relatively few faunistic elements. It is therefore noteworthy that this group of parasites, even with low specificity, will survive in a young ecosystem as this group of trematodes can utilize molluscs as both the first and second intermediate host, transforming into metacercariae in the snails in which they were formed [74].

**Fig. 5:**
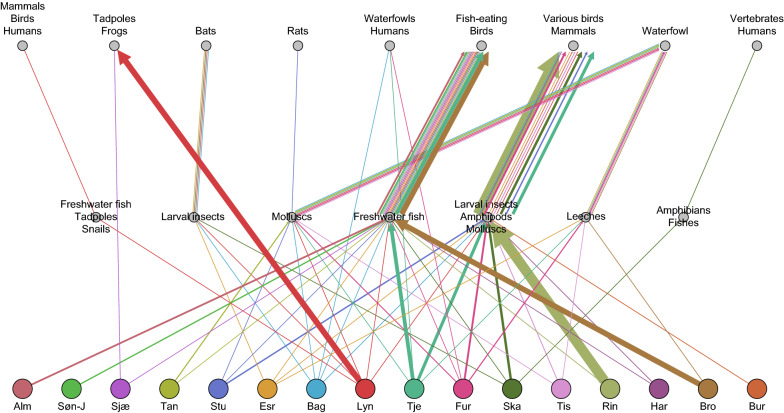
Parasite–host transmission patterns. Note: circles in the middle represent second intermediate hosts in the freshwater environment and circles at the top represent the definitive host groups. The lines indicate trematode species utilizing individual transmission pathways, with the thickness of the lines being proportional to the number of trematode species utilizing each transmission pathway. See Table 1 for definition of lakes

The snail *R. balthica* harbored the most species-rich trematode faunas, with the trematode family Diplostomidae contributing the most. Members of this family group utilize freshwater fish as the second intermediate host and fish-eating birds as the definitive host (Additional file 2: Table S1). BrommeLille sø is a well-known lake hosting a rich teleost fauna. Correspondingly, the parasite fauna showed the strongest relationship with freshwater fish, emphasizing that species richness of trematodes in a snail population relies on the ecology of the freshwater habitat [75]. BrommeLille sø is a shallow lake, with a water depth of 1.5 m [76], thereby providing optimal conditions for an efficient transmission between birds, freshwater fish and snails. This was also reflected by the positive relationship between bird species richness and trematode richness in an estuarine snail in a coastal wetland [77]. In our investigation of another lake, Tissø, a well-known bird habitat in Denmark, trematodes utilizing larval insects as the second intermediate host and piscivorous birds as the definitive host were dominant (Fig. 5). Also Furesø, a large waterfowl habitat, is characterized by the snail parasites using birds as final hosts. Tjele langsø harbors parasites that reflect a dominant representation of freshwater fish, and in Lyngby sø, the occurrence of tadpoles and frogs was reflected by the dominant representation of specific trematodes. It has been argued that parasite diversity is positively related to host diversity [78], and based on theoretical predictions, host and trematode species abundance has been found to be correlated [79]. In our study, Pearson’s correlation analysis between these factors revealed a non-significant positive correlation (*r* = 0.4245, *P* = 0.1012). It has also been suggested that a high diversity of snail hosts in itself is not the driving factor of trematode species richness; rather, it is the particular composition of snail species present, the abundance of those species and the degree of host specialization among digeneans which determine the trematode species richness [34]. In this context, it is worthwhile to note that we confirmed that *L. stagnalis* and *R. balthica* can be regarded as ‘key host species’ hosting the main proportion of the parasite species present [80]. In our study, the prevalence of infection in August and September was relatively high, compared to the spring time, which indicates that temperature could promote the transmission of parasites and increase their local abundance [81]. A relatively high snail and hosts species abundance was found in Furesø, Bagsværd sø and Esrum sø, whereas Ringen sø, Tissø, BrommeLille sø and Tjele langsø contain few species. Species richness may in that case be influenced by short distances between the ponds/lakes, resulting in additional dispersal modes, which in turn results in high immigration rates and/or low extinction rates [30].

## Conclusions

In this work, we have proposed a method for supplementing biological surveys of Danish freshwater systems. By investigating the occurrence of snail-borne trematodes released as cercariae in 21 freshwater lakes in Denmark, we show the presence of additional hosts in the habitats. Using this approach, we found that trematodes released from the snail host as cercariae may act as bioindicators that elucidate the ecology of the investigated freshwater habitat. We confirmed that *L. stagnalis* and *R. balthica* can be regarded as ‘key host species’ hosting the main proportion of the parasite species present. The presence of the parasites in a certain freshwater system indicates the existence of other host organisms that act as secondary and final hosts for the parasites. The presence of these hosts may have implications for fish health in the ecosystem due to the pathogenicity of the trematodes, such as eye flukes, heart flukes and blood flukes, that parasitize fish. We also recovered three zoonotic trematode species from the snail species *L. stagnalis*,* R. balthica* and *S. palustris,* in four different lakes. This finding emphasizes that monitoring of trematode infections in snails may be applied as a tool for surveying occurrence of zoonotic parasites with medical importance. The seasonal occurrence of infection suggests that the climate may have a profound influence on the ecosystems. In this paper, we propose a framework for studying trematode–host interactions and association patterns. We therefore advocate for linking molecular data (for precise and easy diagnostics) to an updated ecological situation of any freshwater habitat. Such a tool could be used as a novel and effective prevention system by farms rearing aquatic animals.

## Supplementary Information

**Additional file 1: Fig. S1** Phylogenetic analysis of ITS sequences. Own sequences are shown in red.

**Additional file 2: Table S1** Overall prevalence of the trematode species (% of infected snails in the pooled samples from March to September in 2019) infecting the eight snail species in the 21 lakes. Lakes where prevalence was zero are indicated by a minus (−). Intermediate and definitive hosts are provided based on published data from literature [6, 87–97]. *small sample size.

## Data Availability

The data from the study are available upon publication. All data supporting the conclusion within this article are included within the article. Representative sequences were submitted to the GenBank database under the accession numbers MW000945-MW001148. All raw sampling data are available in Table 2 and Additional file 2: Table S1.
